# Polyphenols: From Theory to Practice

**DOI:** 10.3390/foods10112595

**Published:** 2021-10-27

**Authors:** Alberto Bertelli, Marco Biagi, Maddalena Corsini, Giulia Baini, Giorgio Cappellucci, Elisabetta Miraldi

**Affiliations:** 1 Department of Biomedical Sciences for Health, University of Milan, 20133 Milan, Italy; alberto.bertelli@unimi.it; 2 Department of Physical Sciences, Earth and Environment, University of Siena, 53100 Siena, Italy; marco.biagi@unisi.it (M.B.); baini3@student.unisi.it (G.B.); cappellucci@student.unisi.it (G.C.); 3 Department of Biotechnology, Chemistry and Pharmacy, University of Siena, 53100 Siena, Italy; maddalena.corsini@unisi.it

**Keywords:** polyphenols, biological properties, antioxidant and antiviral activity, bioavailability, preclinical data

## Abstract

Background: The importance of polyphenols in human health is well known; these compounds are common in foods, such as fruits, vegetables, spices, extra virgin olive oil and wine. On the other hand, the different factors that modulate the biological activity of these compounds are less well known. Conceptualization of the work: In this review we took into account about 200 relevant and recent papers on the following topics: “polyphenols bioavailability”, “polyphenols matrix effect”, “food matrix effect”, “polyphenols-cytochromes interaction”, after having reviewed and updated information on chemical classification and main biological properties of polyphenols, such as the antioxidant, anti-radical and anti-inflammatory activity, together with the tricky link between in vitro tests and clinical trials. Key findings: the issue of polyphenols bioavailability and matrix effect should be better taken into account when health claims are referred to polyphenols, thus considering the matrix effect, enzymatic interactions, reactions with other foods or genetic or gender characteristics that could interfere. We also discovered that in vitro studies often underrate the role of phytocomplexes and thus we provided practical hints to describe a clearer way to approach an investigation on polyphenols for a more resounding transfer to their use in medicine.

## 1. Introduction

Polyphenols are natural compounds synthesized exclusively by plants, with chemical features related to phenolic substances with reported bioactivities to modulate oxidative and inflammatory stress, to alter macronutrient digestion and to exert prebiotic-like effects on gut microbiota.

Polyphenols are almost ubiquitous in plants, being generally involved in the attraction of pollinators, the execution of structural functions, the defense against ultraviolet radiation and the protection of plants against microbial invasion and herbivores [1,2,3].

These compounds are also common in dietary, such as fruits, vegetables, nuts, seeds, flowers and tree barks and common beverages such as wine, beer and tea and are, therefore, an integral part of the human diet. They are partially responsible for the sensory and nutritional qualities of plant foods, for example astringency, color and odor depending on the content of polyphenolic compounds [4]. Furthermore, some can also bind and precipitate macromolecules, such as dietary proteins, carbohydrates and digestive enzymes, thereby reducing food digestibility [5].

Surely, these compounds attained the prominent position due to their wide distribution in plant-based foods and significant evidence of negative correlation of their consumption with cancers, diabetes and cardiovascular diseases. Both epidemiological and clinical evidence suggest that diets high in polyphenols can reduce risk of several age-related chronic diseases [6]. In this context, there has been a significant increase in the number of studies related to the application of components with functional properties and compounds from natural sources in different types of foods, with a view to creating differentiated products with high added value [4,7,8,9].

In this review we primarily aimed to focus some pivotal, but often underrated aspects of polyphenols related with their role in health maintenance; in detail, we called into the question bioavailability limits and metabolic interactions of this large class of secondary metabolites. We also attempted to challenge the issue of scarce absorption of polyphenols and the role of phytocomplexes in understanding strengths and limits of in vitro studies, providing practical hints to better approach the investigation on the topic.

We took into account about 200 relevant and recent papers on specific topics such as polyphenols bioavailability, polyphenols matrix effect, food matrix effect, polyphenols-cytochromes interaction, whereas the most impacting and recent literature on chemical and biological properties was comprehensively reviewed, by searching in vitro, in vivo and clinical studies.

## 2. Polyphenols: Chemical Structure and Biosynthesis

The chemical structure of polyphenols is characterized by the presence of at least one phenyl rings and one or more hydroxyl substituents. Phenolics range from simple small single aromatic-ring structures to the complex and weighty condensed tannins. Polyphenols originate in nature through two main pathways that can occur independently or together [10]. 

One route involves the binding of two-carbon units, that is, activated acetate, to form polyketides, which undergo subsequent cyclisation into polyphenols [5].

**Figure 1:**
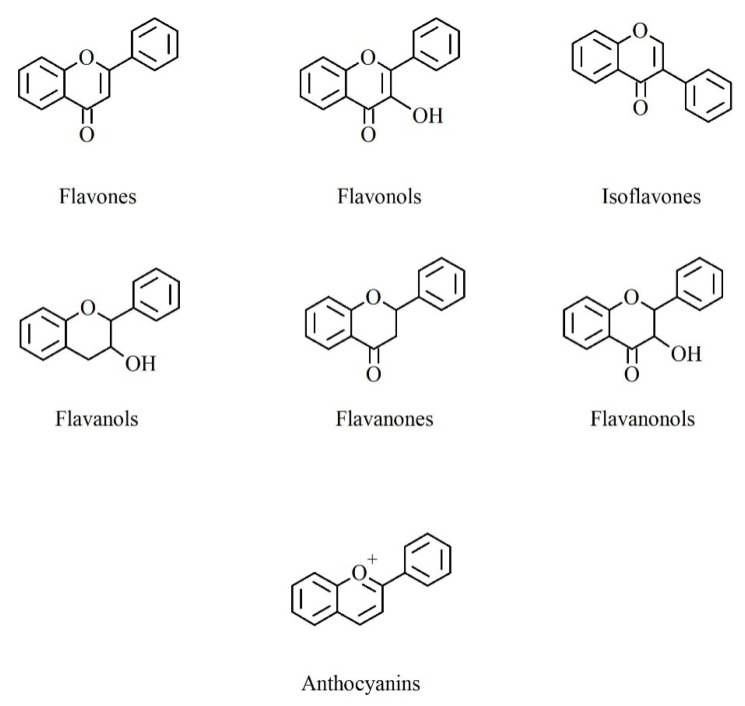
Basic structure of the main different subclasses of flavonoids.

Another mechanism is the shikimic acid pathway, by which most phenolic compounds are biosynthesized. Via this route, the derived carbohydrate precursors of the glycolysis and pentose phosphate pathways are converted to aromatic aminoacids, such as phenylalanine, tyrosine and tryptophan [11]. The enzyme phenylalanine ammonia lyase, via cinnamic acid, gives rise to the formation of caffeic and ferulic acids, which are precursors of the largest group of polyphenols, that is flavonoids. The structural diversity of flavonoid molecules arises from variations in hydroxylation pattern and oxidation state of the central pyran ring, resulting in a wide range of compounds: flavanols, anthocyanidins, anthocyanins, isoflavones, flavones, flavonols, flavanones and flavanonols [10] (Figure 1).

There are many ways to classify polyphenols. The simplest of which is the subdivision into flavonoids and non-flavonoids, but they can also be subdivided into many subclasses depending on the number of phenol units within their molecular structure, substituent groups and/or the linkage type between phenol units.

## 3. Polyphenols: Not Only Conventional Antioxidants

**Figure 2:**
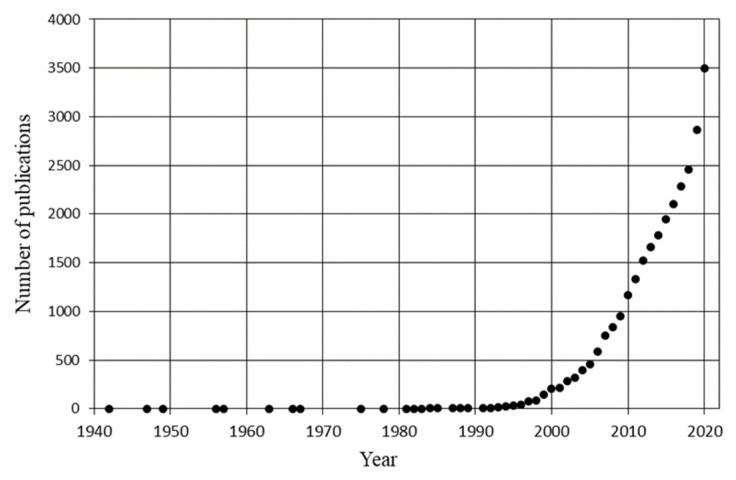
Increase in the number of publications regarding polyphenols and antioxidants in the past 100 y. Publications are those registered in the Scopus database (October 2021). Results retrieved from the query “Polyphenols” and “Antioxidant”.

Despite their wide distribution in plants, the antioxidant effects of polyphenols have come to the attention of scientific community only rather recently. Research on antioxidant properties of polyphenols truly began after 1995 (Figure 2), as the number of papers published/year attests. Perhaps, the main factor that has delayed research on polyphenols is the considerable diversity and complexity of their chemical structures. 

Analytical methods used for their quantification and biological data obtained for polyphenols, are dispersed in a manifold of literature sources. This is complicated by the fact that different polyphenol content in each food may vary greatly according to variety, agricultural and storage conditions. Due to the complexity of this wide group of plant metabolites, however, many polyphenols remain unidentified. As a result, information in the literature on the content and composition of polyphenols in plant foods is not only incomplete, but also contradictory and difficult to compare. Some efforts are spent to organize this multitude of information in databases, such as Phenol-Explorer that provides detailed information on the classes and distribution of polyphenols in foods [12].

The role of dietary polyphenols in maintaining health and in disease prevention is unquestionable and has been attributed, in part, to the antioxidant properties and in part to the free radical-scavenging capacity of these biomolecules. The total antioxidant activities of fruits is mainly due to their polyphenols content, other than vitamin C [13] since they suppress the generation of free radicals and have the role of chain-breakers in the direct radical scavengers of the lipid peroxidation chain reactions [14].

Beyond to antioxidant and radical scavenging ability, polyphenols are also known as metal chelators. In fact, the presence of aromatic rings in conjunction with the occurrence of some functional groups (carboxyl, hydroxyl and carbonyl groups) making them able to bind to different metals [15]. This ability is important for plants, because phenols enhance nutrients uptake by forming chelates with metal ions. Moreover, chelation of transition metals such as iron or copper, reduces the rate of Fenton reaction, thus preventing oxidation caused by reactive hydroxyl radicals [16].

Furthermore, it has been found that polyphenol scan function as co-antioxidant and are involved in the regeneration of essential vitamins. As an example, Zhou and co-authors [17] reported a detailed study on the mechanism of the antioxidant synergism of α-tocopherol with green tea polyphenols. The α-tocopherol is the principal component and the most active form of vitamin E and it is the major endogenous lipid-soluble chain-breaking antioxidant in human plasma. The antioxidant efficiency of vitamin E could be enhanced by another coexisting antioxidant (such as vitamin Cand green tea polyphenols) if the latter could reduce the α-tocopheroxyl radical to regenerate vitamin E. Therefore, the α-tocopherol regeneration reaction by coexisting antioxidants plays a crucial role in enhancing the antioxidant efficiency of α-tocopherol and eliminating the so-called tocopherol-mediated peroxidation.

Recently, there is a new point of view from which phytochemicals and particularly flavonoids, do not act as strictly as conventional antioxidants, but also as modulator in cell signaling [18,19]. The comprehension of the mechanism of action of these biomolecules should be the focus of future research on polyphenols.

## 4. The Problem of Bioavailability of Polyphenols

In studies of polyphenols in food, particularly in fruit and vegetables, much effort is put into their identification, but two fundamental characteristics are often overlooked: their effective bioavailability and the fact that these compounds are part of a matrix, the phytocomplex, with other molecules that can interact with each other in unpredictable ways.

The bioavailability of natural molecules depends on several factors such as: interaction with the herbal matrix, the chemical and physical characteristics of the compound, the stability of the digestive process, their metabolization by intestinal enzymes, liver and intestinal microbiota [20,21]. Regarding the bioaccessibility of polyphenols, it can be influenced by the transformation and cooking processes that the food undergoes, the interaction with components of the food matrix, as well as by the food bolus and the fluids secreted by the gastrointestinal tract during the digestion process [20].

Studies conducted on anthocyanins (typical of red wine, red fruits and red onion) have found their low bioavailability: in fact, only 1–2% of anthocyanins introduced with food maintain their original molecular structure. This is due to various factors such as pH variation in the gastrointestinal tract, hydrolytic reactions by enzymes in the small intestine, phase II metabolization processes in the intestine and liver (glucuronidation, sulfation and methylation) and the enzymatic and catabolic action of the intestinal microbiota [22].

In plant foods, the flavonoid family is the predominant one, the constituents of which are mostly found in glycosidic form. Among these, quercetin represents the most abundant constituent. In fact, its presence appears to be high in many foods such as red onions (65 mg/100 g) and cranberry (149 mg/100 g) [23]. The binding with glucose or other sugars confers higher bioavailability to quercetin and flavonols, but also to several other polyphenols: indeed, glycosides can be transported into the enterocyte through the sodium-dependent glucose transporter SGLT1 and subsequently hydrolyzed into cells by a cytosolic β-glucosidase [24]. Some exceptions occur: the bioavailability of flavan-3-ols, typical of cocoa and of green tea [25] is higher than that of other flavonoids; it ranges from 2% to 15% in green tea [26] and from 5% to 10% for cocoa catechins [27]. Dietary flavan-3-ols are among the few flavonoids that are found mainly in an aglyconic form and they are almost stable in the acidic environment of the stomach, while they are less stable in the alkaline intestinal pH [26]. The bioavailability of flavan-3-ols is closely related to their chemical structure, to pH change and strongly influenced by the intestinal microbiota [28,29].

Stilbenes belong to the group of non-flavonoid polyphenols: resveratrol is the most investigated compound of this class, found in foods such as grapes, peanuts, berries and red wine [30]. Resveratrol is characterized by a poor pharmacokinetic profile as it has low water solubility, low chemical stability during the digestive process and consequently low bioavailability, although it has been attributed important positive biological activities for human health [31].

It seems that the reduced oral bioavailability of resveratrol is caused by its susceptibility to undergo sulfation and glucuronidation during phase II reactions in the gut and in the liver [32], as well as extensive metabolism by gut bacteria [33]. It was observed that after oral consumption of 25 mg resveratrol, less than 10 ng/mL of plasmatic peak concentration of resveratrol after 0.5 h was achieved [34,35,36,37].

Very recently, Kamiloglu and co-authors [38] reviewed the effect of food matrix on flavonoids bioavailability and through a comprehensive analysis of in vitro, in vivo and clinical studies showed that different classes of common dietary flavonoids are markedly influenced in their absorption by macro- and micronutrients. In particular, authors discussed that flavonoids such as catechins, antocyanidins, oligomeric proanthocyanidins, tannins, flavones, flavonols, in different measures, have higher bioavailability in particular in the presence of oils, lipids and carotenoids, but also when combined with digestible carbohydrates, hydrophilic and lipophilic vitamins, alkaloids such as caffeine and P-glycoprotein inhibitors such as piperine, curcumin. On the contrary, in vitro and in vivo findings suggested that minerals and in a higher extent proteins and dietary fibers negatively affect flavonoids absorption.

Drakou and co-authors [39] also investigated how iron and zinc absorption (dialyzability) could be influenced by food matrix in an in vitro digestion model, taking into account different varieties of selected foods, from conventional or organic farming, namely table olives, tomatoes preparations and legumes containing different amounts of polyphenols. Authors found that differences in iron and zinc dialyzability were observed among different varieties of table olives, tomatoes and legumes tested and not from farming conditions or polyphenols content.

Little is known about the interactions between different polyphenols at pharmacokinetic level. In [38] variable interactions, positive and negative, were reported, dependent on the used experimental model. In humans, findings suggest a positive effect of the polyphenols matrix: for examples, when administered together, quercetin positively modulates resveratrol pharmacokinetic features; indeed, quercetin inhibits liver glucoronidation and sulphation of resveratrol, increasing its bioavailability [40,41]. Researches on extra virgin olive oil (EVOO) helped to clarify, at least in part, the intricate interactions, synergies, or interferences of polyphenols. The two most studied phenols in EVOO are tyrosol (TYR) and hydroxytyrosol (HT), endowed with antioxidant, anti-inflammatory and cardioprotective properties [42] that are bio-transformed by CYP2A6 and CYP2D6 both in animal models [43] and in humans [42]; TYR is converted in HT and this may lead to a beneficial effect as HT would appear to be more active than TYR. The same positive effect of TYR/HT transformation can be obtained in red wine and dark beer [44]. CYP2A6 and CYP2D6 activity differ in male and female, thus indicating that not all individuals metabolize phenols in the same way and also highlighting differences between the two sexes [44]. Other examples worthy to be cited in terms of higher bioavailability of dietary polyphenolic complexes are yet cited red wine for resveratrol absorption [35] and flavan-3-ols of green tea [26,45] and cocoa [27].

When considering the phytocomplex, enzymatic interferences between different polyphenols and between polyphenols and food or drugs should be carefully considered.

Cytochrome interactions are the best studied enzymatic metabolic interferences of polyphenols. Interestingly, this has been recently reported for epigallocatechin-3-gallate (EGCG), a polyphenol abundant in green tea [46]: EGCG’s modulates activity of cytochromes P450 (CYP) 1A2, 2E1 and 3A4, showing anti-inflammatory and protective activity against potentially hepatotoxic drugs.

Other biological health-protective activities resulting from the modulation of cytochromes by polyphenols could also be mentioned: resveratrol (CYP1A1-CYPB1) as a potential chemopreventive against dioxin induced human mammary carcinogenesis [47], ε-viniferin (grape-wine) as an inhibitor of CYP1A1, CYP1B1 and CYP1E1, cytochromes involved in the activation of carcinogenic compounds [48] and even red wine (CYP2E1) in the mitigation of ethanol damage in the kidney [49].

However, potentially undesirable effects of modulation of cytochromes that may lead to their partial inhibition or inactivation should not be neglected: for example, 6′-7′ of hydroxybergamottin and bergamottin (CYP2C19) in grapefruit juice [50] and resveratrol (CYP1B1-CYP1A1-CYP1A2) [51], effects also confirmed in humans [52].

Littlewood and co-authors [53] reported the activity of red wine extracts on phenolsulfotransferases and showed, probably due to the action of phenolic flavonoids, an inhibition on human platelet P- and M-phenolsulfotrnasferases of 99% and 12%, respectively. Inhibition of these two enzymes, involved in the metabolism of many phenols and also drugs, could have important clinical consequences. Maier-Salamon and co. [54] also studied biotransformation provided by uridine 5′-diphospho-glucuronosyltransferases (UDP-glucuronosyltransferases) on grape polyphenol piceatannol reporting that glucoronidation strongly influences its bioavailability, resulting to be crucial in the elimination of orally taken dietary piceatannol [55]. Even when it comes to EVOO, research continues with the discovery of new phenols such as oleocanthal [56,57,58] or oleacein (oleuropeina-glycone) [59].

Table 1 summarizes aspects of bioavailability of main different class of dietary polyphenols, cytochrome interactions and the matrix effect.

One should therefore wonder how a consumer could be sure to get a beneficial effect from dietary polyphenols without considering that, enzymatic interactions, reactions with other foods or genetic or gender characteristics could interfere. Therefore, the study of the biological activities of polyphenols still remains a challenge, because if it is true that: “In the foodomics era, considering a complex foodome including over 25,000 substances that make up the human diet, it appears to be outdated to pursue the hunt for biological activities of one function/compound at the time” [66], it is equally true that: “Studies that identify the active components of foods along with the mechanisms by which they exert their effects, may not only overshadow speculation, but should improve our understanding of the importance of diet and may also accelerate the identification of new anticancer agents” (http://oncology.thelancet.com, last accessed on 20 September 2021).

Time will tell which of these two research conceptions is the more effective.

## 5. Bioavailability of Polyphenols: What In Vitro Tests Do Not Tell Us

Important concerns related to the poor bioavailability of polyphenols and matrix effect are often underrated aspects and in vitro studies, that count over than 90% of overall studies on polyphenols, may lead to unplausible perspectives in the context of the role of polyphenols for human health.

Studies on antioxidant and anti-inflammatory properties of polyphenols clearly depict the difference between in vitro data and the clinically observed effects of polyphenols.

Many different polyphenols, both considered as dietary products and active principles of medicinal plants used in prevention and in treatment of diseases, have been claimed to display a strong antioxidant activity. The importance of counteracting oxidative stress is pivotal in many conditions characterized by a red/ox unbalance, such as aging, chronic inflammatory diseases, but also cancer and degenerative conditions [67]. In vitro studies have highlighted the high biological potential of many polyphenols and attempted to assess different mechanism of action, focusing on scavenging activity and modulation of intracellular antioxidant enzymes. IC_50_ of different polyphenols in DPPH (2,2-diphenyl-1-picrylhydrazyl) or ABTS (2,2′-azino-bis(3-ethylbenzothiazoline-6-sulfonic) acid) essays have been collocated in the range of 5–25 μg/mL, as in the case of tyrosol [68], hyperoside [69], EGCG [70], cyanidin [71] and higher for resveratrol [72]. These concentrations are higher than those achievable in vivo when the same polyphenols have been tested and it could be postulated that in vivo direct scavenger activity and indirect interaction with antioxidant intracellular pathways participate at the same time in the biological effect of polyphenols.

As recently reviewed by Abdel-Tawab in 2021 [73] about the anti-inflammatory effect of well-known natural products—among those polyphenols such as curcumin, quercetin and resveratrol—many questions should be better discussed and in vitro data should be accurately interpreted. Curcumin is a well-known anti-inflammatory molecule with a elucidated mechanism of action that takes into account the upstream and downstream interaction with several pathways such as lipo- and cyclo-oxygenase modulation (LOX and COX), mitogen activated protein kinases (MAPKs) and NF-κB signaling (https://www.ema.europa.eu/en/documents/herbal-report/final-assessment-report-curcuma-longa-l-rhizoma-revision-1_en.pdf, last accessed on 20 September 2021); nevertheless, in cell models the effectiveness of curcumin has been only recorded at high concentration and IC_50_ in COX-2 and NF-κB inhibition are >50 μM and 10–20 μM, respectively [73], concentrations unreachable after oral administration even of improved liposomial formulations containing all the pool of curcuminoids, i.e., curcumin, demethoxycurcumin and bisdemethoxycurcumin, that could provide 200 ng/mL ca. as maximum plasmatic concentration of total curcuminoids [74]. For these reasons, even if different specific targets have been proposed to explain anti-inflammatory effect of curcumin, clinically observed effects of this polyphenol have unclear mechanism.

Similarly, in vitro quercetin showed marked inhibitory effects on different inflammatory targets such as ERK and p38 MAPKs, NF-κB and it showed to reduce pro-inflammatory IL-1β, IL-6, IFN-γ and TNF-α cytokines in lipopolysaccharide (LPS)-stimulated immune cells; effective concentrations of quercetin in targeting inflammatory targets in in vitro studies ranged from 1 to 25 μM [64,65,66,67,68,69,70,71,72,73,74,75]. Despite this, again, the poor oral bioavailability of this flavonoid, not more than 2% in the aglycone form [68], lead us to consider that only repeated administration of high dosage of quercetin could exert some effects related to the interaction with inflammatory targets.

Indeed, recently Dehghani and co-authors [76] showed that 500 mg/day of quercetin for 8 weeks modulated antioxidant markers, but failed in reducing inflammatory ones in post-myocardial infarction patients, whereas a previous clinical trial [77] reported that the same dosage of 500 mg/day of quercetin for 8 weeks in women with rheumatoid arthritis significantly reduced TNF-α level in comparison to placebo. It is known that dietary quercetin, in glycoside form, is more bioavailable than aglycone (up to 17%) [64], but the amount of this flavonoid in food is low. Red onion is the main conventional dietary source of quercetin glycosides (0.65 mg/g) [23], but clinical trials failed to address any evident beneficial role of onions and concentrated extracts in men [64,78].

As regards resveratrol, this stilbene has been extensively investigated both in vitro and in clinical trials. Inflammatory targets targeted by resveratrol and effective concentrations found in vitro cell models were similar to those depicted for quercetin [73] but also for resveratrol, bioavailability is not more than 1% of oral dosage administered [34] and many mechanisms elucidated in vitro are not unequivocally connected with plausible in vivo biological effects. As seen for quercetin, and similar for resveratrol, a long period of administration (4–12 weeks) with 500–1000 mg/day is required to produce a marked modulation of inflammatory markers, in case of patients with ulcerative colitis [79], in patients with polycystic ovary syndrome [80] and in the management of rheumatoid arthritis [81].

Evidence have claimed that plausibly quercetin and resveratrol metabolites, such as sulfonated and glucuronidated derivatives in vivo may have a major role, higher than the unmodified bioavailable fraction [82].

If in vitro assays may lead to improper understanding of mechanism of action of polyphenols, in some cases, because the poor bioavailability of these natural products, also they could propose unplausible biological effects. It is the current state of the research on antiviral activity of polyphenols [83], with particular regard on the activity against SARS-CoV-2. To date, more than 110 papers have been published in only 2 years to describe the potential of polyphenols as agents against the new SARS-CoV-2 in vitro and a similar number of computational studies were performed; again, resveratrol, curcumin, EGCG and quercetin emerged as promising molecules. These dietary polyphenols have clearly showed specific inhibitory activity on the angiotensin converting enzyme 2 receptor (ACE2r)–viral spike protein binding [84,85,86], and/or on viral proteases [87,88], but in any cases IC_50_ was below 1 mM. One of the lowest virucidal effect against SARS-CoV-2 as regards dietary polyphenols was reported for curcumin (IC_50_ 0.448 mM), mainly exerted through a non-specific virucidal mechanism [87]. These data once more suggest that if a beneficial role for dietary polyphenols could be postulated in pre- or post-viral entry, it should be proved through clinical trials that currently are missing since pharmacokinetic aspects and matrix interferences have been very rarely considered and in vitro studies are inadequate to draw any conclusion.

## 6. From the Bench to Pre-Clinical and Clinical Studies on Polyphenols: Practical Instructions for Use

Cell models and other in vitro studies represent a pivotal step to study natural compounds and other active principles; ethical, versatile and cost effective, they are a step necessary to move forward in every field of pharmacology. Thus, is it possible to treasure what animal models and clinical trials taught as regards polyphenols to plan more sounding in vitro preliminary studies? Definitively yes. Some simple suggestions, very often underrated, should be considered.

### 6.1. Single Polyphenols or Phytocomplex: The Importance of the Sample under Investigation

The study of a polyphenolic compound should start from the knowledge of its natural occurrence, market availability and, in case, matrix effect. Quercetin, curcumin or resveratrol could be purified from dietary sources and they are easily available as extracts with a content >95–98%, whereas ECGC and other catechins, anthocyanidins, the most part of other flavonoids or phloroglucinols are only available, in variable amounts, in herbal extracts or in other concentrated forms. In these cases, if a molecule is investigated as a pure compound in in vitro tests, its actual content in extracts or preparations as well as the matrix effect should be always considered. Practically, the study of compounds such as EGCG or malvidin or hyperforin, just to cite well known and investigated molecules, in cell models and cell-free tests should be compared to actual available sources, in these cases, green tea, red fruits and *Hypericum perforatum* L. extracts, in order to better interpret plausible biological effects.

### 6.2. Pharmacokinetic Aspects

The knowledge of the pharmacokinetic of polyphenols is fundamental not only to plan in vitro experiments testing plausible concentrations, but also to avoid upstream methodological errors: for example, many papers have been published on several biological effects exerted by high concentrations of hyperoside and genistin in cell models, despite these two glycosylated polyphenols undergo a rapid and extensive metabolism produced by gut flora, their absorption is even lower than aglycone forms [89,90] and they are converted in quercetin and genistein that should have been tested as well.

The use of computational tools such as ADMET predictors provides a simple and modern approach in preclinical studies [91], which is particularly useful for the study of natural compounds and for setting the correct concentrations and set of molecules to be tested.

### 6.3. Study of the Mechanism of Action

This is a point yet discussed in this paper, but worthy to be highlighted: in vitro studies performed at concentrations achievable after oral administration provide fundamental understanding of mechanism underlying observed biological effects of polyphenols and other active principles, but the opposite, i.e., transferring in vitro results to assume a biological effect is very often a confounding factor as seen in many papers on curcumin, resveratrol and other polyphenols.

Moreover, even practical experimental elements result in an in vitro investigation on mechanism of action plausible or unrealistic. An example that could be cited is the duration of treatment used: upstream binding on surface receptors, transduction factors phosphorylation and activation and many antiradical and antioxidant effects should be studied only for a very short time, in terms of minutes, whereas the downstream release of cytokines or other cell mediators could be investigated after a longer time of treatment.

Table 2 summarizes the main concerns related to in vitro studies of common dietary polyphenols with poor bioavailability studied as inflammatory modulators and practical suggestions to overcome them.

## 7. Conclusions

### Polyphenols: A Lesson from Pharmacokinetics to Transfer Theory to Practice

The research on polyphenols covers a wide space within the scientific community, ranging from agriculture to applied botany, from nutrition to evidence-based medicine. It is unambiguous that polyphenols consumption, both from dietary and from concentrated extracts, is related to many positive effects on human health, starting from the modulation of oxidative stress and inflammation. Paradoxically, positive epidemiologic data and clinical outcomes have often led to an uncritical acceptance of mechanisms and classical pharmacological activities attributed to polyphenols, but actually several complicating elements have to be taken into account. In this review, some of these factors have beenfocused on. Pharmacokinetic aspects of polyphenols and metabolic interactions are called into question; indeed, it could be always questioned if and in what extent a beneficial effect from polyphenols could be obtained without considering the matrix effect, enzymatic interactions, reactions with other foods or genetic or gender characteristics that could interfere. It is true that, particularly with food, pursuing the biological activities of only one compound is still not convincing. Underrating the role of phytocomplexes and bioavailability in studying polyphenols is a common feature of many in vitro studies, the most of those published on the topic, but it is an actual confounding factor for the understanding of polyphenols biological effects. Here, we attempt to give practical hints and to describe a clearer way to study polyphenols on the bench for a more resounding transfer to clinical trial and use in medicine.

## Figures and Tables

**Table 1:** Oral bioavailability of the most common dietary polyphenols, their interactions with different CYP450 isoforms and the effect in terms of modulation of bioavailability of polyphenols exerted by carbohydrates, lipid, proteins, minerals and other nutrients and food minor constituents.

Polyphenol or Polyphenol Class	Oral Bioavailability	Main Cytochrome Interactions	Polyphenol-Polyphenol Interaction	Nutrients Interaction
Anthocyanidins	1–2% [22]	Weak CYP450 inhibitors [60]	Not known	Lipids, carotenoids, digestible carbohydrates, hydrophilic and lipophilic vitamins, alkaloids, P-glycoprotein inhibitors improve flavonoids and curcumin bioavailability Minerals, proteins and dietary fibers decrease flavonoids bioavailability [38]
Curcumin	<1% [61]	CYP3A4 (inhibition) [38]	Not known	
Flavan-3-ols	2–15% in green tea; 5–10% in cocoa beans [26,27]	EGCG: inhibition of the activity of CYP1A2 CYP3A4 CYP2E1 [46]	Green, black and oolong tea phenolic complex improve EGCG bioavailability [45]	
Hydroxytyrosol	High [62]	Plausible interaction with CYP450 [62]	In olive oil tyrosol is converted in hydroxytyrosol by CYP2A6 and CYP2D6 [42,43]	
Isoflavones	High [63]	Genistein: CYP450 ω-hydroxylase subfamily inhibitor [60]	Not known	
Quercetin	<1% (up to 17% when ingested as glycoside) [64]	CYP1A2 CYP2A6 (inhibition) [65]	Not known	
Resveratrol	<1% [37]	CYP3A4 CYP1B1 CYP1A1 CYP1A2 (inhibition) [31,37,51]	Red wine phenolic complex improves resveratrol bioavailability [35,37] Quercetin improves resveratrol bioavailability [40,41]	

**Table 2:** In vitro anti-inflammatory activity of quercetin, curcumin and resveratrol: concerns and suggestions for a proper study of these poor available polyphenols.

Polyphenol	Studied Effects	Models	Findings	Main Concerns	Possible Suggestions
Quercetin	Pro-inflammatory cytokines release inhibition Cyclooxygenase and lipoxygenase inhibition Inhibition of Src- and Syk-mediated PI3K-(p85) Inhibition of intracellular calcium influx and PKC signaling	Human umbilical cord blood-derived cultured mast cells (hCBMCs) Human normal peripheral blood mononuclear cells (PBMC) Human monocytes (THP-1) RAW 264.7 macrophages T lymphocytes Mast cells Microglial cells BV-2	Anti-inflammatory activity only exerted at concentrations >1 μM, more often in the range 10–100 μM [73,75]	Effective concentrations are high if compared with those normally achievable in vivo [73] Quercetin is considered one of the most impacting dietary flavonoids, but it mostly occurs in food as glycoside [23] In vitro demonstrated effects could only be referred to repeated administration of high dosesof quercetin [76,77]	Quercetin should be tested in vitro at nanomolar level Quercetin should be investigated both as single compound and in matrix when its dietary role is taken into account Investigation on quercetin should consider simulated digestion in order to evaluate the role of metabolites
Curcumin	Upstream signaling and modulation of transduction and transcription factors Downstream level of pro-inflammaotry markers	Different human immune cell lines Human umbilical vein endothelial cells (HUVEC) Tracheal smooth muscle cells Head and neck cancer cells RAW 264.7 macrophages Oesophageal epithelial cells Microglial cells	Strong anti-inflammatory activity exerted at concentrations >10 μM [73,92]	Effective concentrations are high if compared with those normally achievable in vivoand in vitro tests hardly could explain clinical findings [73] Curcumin occurs in food and food supplements in complex with other curcuminoids [23]	Curcumin and curcuminoids should be tested in vitroat nanomolar level Curcumin should be investigated both as single compound and in matrix together with other curcuminoids
Resveratrol	Arachidonic acid pathways MAPKs pathways NF-κB signaling AP-1 pathways Pro-inflammatory cytokines release inhibition	A549 adenocarcinomic human alveolar basal epithelial cells Human keratinocytes Human mammary epithelial cells Human T lymphocytes THP-1 HUVEC RAW 264.7 macrophages Myeloid leukemia cells Cardiomyocytes Chondrocytes Mesangial cells Osteoblasts Pancreatic cancer cells Benign prostatic hyperplasia epithelial cell line (BPH-1)	Anti-inflammatory activityexerted at concentrations >1 μM [73,93]	Effective concentrations are high if compared with those normally achievable in vivoand in vitro tests hardly could explain clinical findings [73] In vitro effects could be not referred to dietary resveratrol contained in grape, wine or in other source, given its poor content [37,93]	Resveratrol should be tested in vitro at nanomolar level Investigation on resveratrol should consider simulated digestion in order to evaluate the role of metabolites

## Data Availability

Not applicable.

## References

[1] Harborne J.B., Williams C.A. (2000). Advances in flavonoid research since 1992. Phytochemistry.

[2] Manach C., Scalbert A., Morand C., Rémésy C., Jiménez L. (2004). Polyphenols: Food sources and bioavailability. Am. J. Clin. Nutr..

[3] Mukherjee C., Chakraborty S. (2021). Study of dietary polyphenols from natural herbal sources for providing protection against human degenerative disorders. Biocatal. Agric. Biotechnol..

[4] Pandey K.B., Rizvi S.I. (2009). Plant Polyphenols as Dietary Antioxidants in Human Health and Disease. Oxidative Med. Cell. Longev..

[5] Cutrim C.S., Cortez M.A.S. (2018). A review on polyphenols: Classification, beneficial effects and their application in dairy products. Int. J. Dairy Technol..

[6] Debelo H., Li M., Ferruzzi M.G. (2020). Processing influences on food polyphenol profiles and biological activity. Curr. Opin. Food Sci..

[7] Maganha L.C., Rosim R.E., Corassin C.H., Cruz A.G., Faria J.A.F., Oliveira C.A.F. (2013). Viability of probiotic bacteria in fermented skim milk produced with different levels of milk powder and sugar. Int. J. Dairy Technol..

[8] Ribeiro A., Caleja C., Barros L., Santos-Buelga C., Barreiro M.F., Ferreira I.C.F.R. (2016). Rosemary extracts in functional foods: Extraction, chemical characterization and incorporation of free and microencapsulated forms in cottage cheese. Food Funct..

[9] Balthazar C.F., Silva H., Cavalcanti R., Esmerino E., Cappato L., Abud Y., Moraes J., Andrade M., Freitas M., Sant’Anna C. (2017). Prebiotics addition in sheep milk ice cream: A rheological, microstructural and sensory study. J. Funct. Foods.

[10] Singla R.K., Dubey A.K., Garg A., Sharma R.K., Fiorino M., Ameen S.M., Haddad M.A., Al-Hiary M. (2019). Natural Polyphenols: Chemical Classification, Definition of Classes, Subcategories, and Structures. J. AOAC Int..

[11] Abbas M., Saeed F., Anjum F.M., Afzaal M., Tufail T., Bashir M.S., Ishtiaq A., Hussain S., Suleria H.A.R. (2017). Natural polyphenols: An overview. Int. J. Food Prop..

[12] Neveu V., Pérez-Jiménez J., Vos F., Crespy V., du Chaffaut L., Mennen L., Knox C., Eisner R., Cruz J., Wishart D. (2010). Phenol-Explorer: An online comprehensive database on polyphenol contents in foods. Database.

[13] Wang H., Cao G., Prior R.L. (1996). Total Antioxidant Capacity of Fruits. J. Agric. Food Chem..

[14] Rice-Evans C.A., Miller N.J., Paganga G. (1996). Structure-antioxidant activity relationships of flavonoids and phenolic acids. Free. Radic. Biol. Med..

[15] Cherrak S.A., Mokhtari-Soulimane N., Berroukeche F., Bensenane B., Cherbonnel A., Merzouk H., Elhabiri M. (2016). In Vitro Antioxidant versus Metal Ion Chelating Properties of Flavonoids: A Structure-Activity Investigation. PLoS ONE.

[16] Pietta P.-G. (2000). Flavonoids as Antioxidants. J. Nat. Prod..

[17] Zhou B., Wu L.-M., Yang L., Liu Z.-L. (2005). Evidence for α-tocopherol regeneration reaction of green tea polyphenols in SDS micelles. Free. Radic. Biol. Med..

[18] Kostyuk V.A., Potapovich A.I., Suhan T.O., de Luca C., Korkina L.G. (2011). Antioxidant and signal modulation properties of plant polyphenols in controlling vascular inflammation. Eur. J. Pharmacol..

[19] Firuzi O., Moosavi F., Hosseini R., Saso L. (2015). Modulation of neurotrophic signaling pathways by polyphenols. Drug Des. Dev. Ther..

[20] Wojtunik-Kulesza K., Oniszczuk A., Oniszczuk T., Combrzyński M., Nowakowska D., Matwijczuk A. (2020). Influence of In Vitro Digestion on Composition, Bioaccessibility and Antioxidant Activity of Food Polyphenols—A Non-Systematic Review. Nutrients.

[21] Câmara J.S., Albuquerque B.R., Aguiar J., Corrêa R.C.G., Gonçalves J.L., Granato D., Pereira J.A.M., Barros L., Ferreira I.C.F.R. (2020). Food Bioactive Compounds and Emerging Techniques for Their Extraction: Polyphenols as a Case Study. Foods.

[22] Tena N., Martín J., Asuero A.G. (2020). State of the Art of Anthocyanins: Antioxidant Activity, Sources, Bioavailability, and Therapeutic Effect in Human Health. Antioxidants.

[23] Aherne S., O’Brien N.M. (2002). Dietary flavonols: Chemistry, food content, and metabolism. Nutrition.

[24] Hollman P.C., De Vries J.H., Van Leeuwen S.D., Mengelers M.J., Katan M.B. (1995). Absorption of dietary quercetin glycosides and quercetin in healthy ileostomy volunteers. Am. J. Clin. Nutr..

[25] Sánchez-Rabaneda F., Jáuregui O., Casals I., Andres-Lacueva C., Izquierdo-Pulido M., Lamuela-Raventós R.M. (2003). Liquid chromatographic/electrospray ionization tandem mass spectrometric study of the phenolic composition of cocoa (Theobroma cacao). J. Mass Spectrom..

[26] Tenore G.C., Campiglia P., Giannetti D., Novellino E. (2015). Simulated gastrointestinal digestion, intestinal permeation and plasma protein interaction of white, green, and black tea polyphenols. Food Chem..

[27] Flores M.E.J. (2019). Cocoa Flavanols: Natural Agents with Attenuating Effects on Metabolic Syndrome Risk Factors. Nutrients.

[28] Record I.R., Lane J.M. (2001). Simulated intestinal digestion of green and black teas. Food Chem..

[29] Sorrenti V., Ali S., Mancin L., Davinelli S., Paoli A., Scapagnini G. (2020). Cocoa Polyphenols and Gut Microbiota Interplay: Bioavailability, Prebiotic Effect, and Impact on Human Health. Nutrients.

[30] Sirerol J.A., Rodríguez M.L., Mena S., Asensi M.A., Estrela J.M., Ortega A.L. (2016). Role of Natural Stilbenes in the Prevention of Cancer. Oxidative Med. Cell. Longev..

[31] Machado N.D., Fernández M.A., Díaz D.D. (2019). Recent Strategies in Resveratrol Delivery Systems. Chem. Plus. Chem..

[32] Walle T. (2011). Bioavailability of resveratrol. Ann. N. Y. Acad. Sci..

[33] Fu X., Zeng B., Wang P., Wang L., Wen B., Li Y., Liu H., Bai S., Jia G. (2018). Microbiome of Total Versus Live Bacteria in the Gut of Rex Rabbits. Front. Microbiol..

[34] Pannu N., Bhatnagar A. (2019). Resveratrol: From enhanced biosynthesis and bioavailability to multitargeting chronic diseases. Biomed. Pharmacother..

[35] Lee S.Y., Lee S.J., Yim D.G., Hur S.J. (2020). Changes in the Content and Bioavailability of Onion Quercetin and Grape Resveratrol During In Vitro Human Digestion. Foods.

[36] Ragusa A., Centonze C., Grasso M.E., Latronico M.F., Mastrangelo P.F., Sparascio F., Maffia M. (2019). HPLC Analysis of Phenols in Negroamaro and Primitivo Red Wines from Salento. Foods.

[37] Biagi M., Bertelli A.A. (2015). Wine, alcohol and pills: What future for the French paradox?. Life Sci..

[38] Kamiloglu S., Tomas M., Ozdal T., Capanoglu E. (2020). Effect of food matrix on the content and bioavailability of flavonoids. Trends Food Sci. Technol..

[39] Drakou M., Birmpa A., Koutelidakis A.E., Komaitis M., Panagou E., Kapsokefalou M. (2015). Total antioxidant capacity, total phenolic content and iron and zinc dialyzability in selected Greek varieties of table olives, tomatoes and legumes from conventional and organic farming. Int. J. Food Sci. Nutr..

[40] De Santi C., Pietrabissa A., Mosca F., Pacifici G. (2002). Methylation of quercetin and fisetin, flavonoids widely distributed in edible vegetables, fruits and wine, by human liver. Int. J. Clin. Pharmacol. Ther..

[41] De Santi C., Pietrabissa A., Mosca F., Rane A., Pacifici G.M. (2002). Inhibition of phenol sulfotransferase (SULT1A1) by quercetin in human adult and foetal livers. Xenobiotica.

[42] Boronat A., Mateus J., Soldevila-Domenech N., Guerra M., Rodríguez-Morató J., Varon C., Muñoz D., Barbosa F., Morales J.C., Gaedigk A. (2019). Cardiovascular benefits of ty-rosol and its endogenous conversion into hydroxytyrosol in humans. A randomized, controlled trial. Free Radic. Biol. Med..

[43] Rodríguez-Morató J., Robledo P., Tanner J.-A., Boronat A., Pérez-Mañá C., Chen C.-Y.O., Tyndale R.F., de la Torre R. (2017). CYP2D6 and CYP2A6 biotransform dietary tyrosol into hydroxytyrosol. Food Chem..

[44] Soldevila-Domenech N., Boronat A., Mateus J., Diaz-Pellicer P., Matilla I., Pérez-Otero M., Aldea-Perona A., De La Torre R. (2019). Generation of the Antioxidant Hydroxytyrosol from Tyrosol Present in Beer and Red Wine in a Randomized Clinical Trial. Nutrients.

[45] Governa P., Manetti F., Miraldi E., Biagi M. (2021). Effects of in vitro simulated digestion on the antioxidant activity of different Camellia sinensis (L.) Kuntze leaves extracts. Eur. Food Res. Technol..

[46] Yao H.-T., Li C.-C., Chang C.-H. (2019). Epigallocatechin-3-Gallate Reduces Hepatic Oxidative Stress and Lowers CYP-Mediated Bioactivation and Toxicity of Acetaminophen in Rats. Nutrients.

[47] Chen Z.-H., Hurh Y.-J., Na H.-K., Kim J.H., Chun Y.-J., Kim D.H., Kang K.-S., Cho M.-H., Surh Y.-J. (2004). Resveratrol inhibits TCDD-induced expression of CYP1A1 and CYP1B1 and catechol estrogen-mediated oxidative DNA damage in cultured human mammary epithelial cells. Carcinogenesis.

[48] Piver B., Fer M., Vitrac X., Merillon J.-M., Dreano Y., Berthou F., Lucas D. (2004). Involvement of cytochrome P450 1A2 in the biotransformation of trans-resveratrol in human liver microsomes. Biochem. Pharmacol..

[49] Orellana M., Varela N., Guajardo V., Araya J., Rodrigo R. (2002). Modulation of rat liver cytochrome P450 activity by prolonged red wine consumption. Comp. Biochem. Physiol. Part C Toxicol. Pharmacol..

[50] Seki H., Akiyoshi T., Imaoka A., Ohtani H. (2019). Inhibitory kinetics of fruit components on CYP2C19 activity. Drug Metab. Pharmacokinet..

[51] Chang T.K., Chen J., Lee W.B. (2001). Differential inhibition and inactivation of human CYP1 enzymes by trans-resveratrol: Evidence for mechanism-based inactivation of CYP1A2. J. Pharmacol. Exp. Ther..

[52] Offman E., Freeman D.J., Dresser G.K., Muñoz C., Bend J.R., Bailey D.G. (2001). Red wine–cisapride interaction: Comparison with grapefruit juice. Clin. Pharmacol. Ther..

[53] Littlewood J., Glover V., Sandler M. (1985). Red wine contains a potent inhibitor of phenolsulphotransferase. Br. J. Clin. Pharmacol..

[54] Maier-Salamon A., Böhmdorfer M., Thalhammer T., Szekeres T., Jaeger W. (2011). Hepatic Glucuronidation of Resveratrol: Interspecies Comparison of Enzyme Kinetic Profiles in Human, Mouse, Rat, and Dog. Drug Metab. Pharmacokinet..

[55] Miksits M., Maier-Salamon A., Vo T.P.N., Sulyok M., Schuhmacher R., Szekeres T., Jäger W. (2010). Glucuronidation of piceatannol by human liver microsomes: Major role of UGT1A1, UGT1A8 and UGT1A10. J. Pharm. Pharmacol..

[56] Lucci P., Bertoz V., Pacetti D., Moret S., Conte L. (2020). Effect of the Refining Process on Total Hydroxytyrosol, Tyrosol, and Tocopherol Contents of Olive Oil. Foods.

[57] Beauchamp G.K., Keast R., Morel D., Lin J., Pika J., Han Q., Lee C.-H., Smith A.B., Breslin P.A.S. (2005). Ibuprofen-like activity in extra-virgin olive oil. Nat. Cell Biol..

[58] López-Yerena A., Vallverdú-Queralt A., Mols R., Augustijns P., Lamuela-Raventós R.M., Escribano-Ferrer E. (2020). Absorption and Intestinal Metabolic Profile of Oleocanthal in Rats. Pharmaceutics.

[59] Cuyàs E., Verdura S., Lozano-Sánchez J., Viciano I., Llorach-Pares L., Nonell-Canals A., Bosch-Barrera J., Brunet J., Segura-Carretero A., Sanchez-Martinez M. (2019). The extra virgin olive oil phenolic oleacein is a dual substrate-inhibitor of catechol-O-methyltransferase. Food Chem. Toxicol..

[60] Kampschulte N., Alasmer A., Empl M.T., Krohn M., Steinberg P., Schebb N.H. (2020). Dietary Polyphenols Inhibit the Cytochrome P450 Monooxygenase Branch of the Arachidonic Acid Cascade with Remarkable Structure-Dependent Selectivity and Potency. J. Agric. Food Chem..

[61] Kotha R.R., Luthria D.L. (2019). Curcumin: Biological, Pharmaceutical, Nutraceutical, and Analytical Aspects. Molecules.

[62] Bertelli M., Kiani A.K., Paolacci S., Manara E., Kurti D., Dhuli K., Bushati V., Miertus J., Pangallo D., Baglivo M. (2020). Hydroxytyrosol: A natural compound with promising pharmacological activities. J. Biotechnol..

[63] Vitale D.C., Piazza C., Melilli B., Drago F., Salomone S. (2013). Isoflavones: Estrogenic activity, biological effect and bioavailability. Eur. J. Drug Metab. Pharmacokinet..

[64] Li Y., Yao J., Han C., Yang J., Chaudhry M.T., Wang S., Liu H., Yin Y. (2016). Quercetin, Inflammation and Immunity. Nutrients.

[65] Chen Y., Xiao P., Ou-Yang D.-S., Fan L., Guo D., Wang Y.-N., Han Y., Tu J.-H., Zhou G., Huang Y.-F. (2009). Simultaneous action of the flavonoid quercetin on cytochrome p450 (cyp) 1a2, cyp2a6,n-acetyltransferase and xanthine oxidase activity in healthy volunteers. Clin. Exp. Pharmacol. Physiol..

[66] Khakimov B., Engelsen S.B. (2017). Resveratrol in the foodomics era: 1:25,000. Ann. N. Y. Acad. Sci..

[67] Neha K., Haider R., Pathak A., Yar M.S. (2019). Medicinal prospects of antioxidants: A review. Eur. J. Med. Chem..

[68] Tundis R., Conidi C., Loizzo M.R., Sicari V., Cassano A. (2020). Olive Mill Wastewater Polyphenol-Enriched Fractions by Integrated Membrane Process: A Promising Source of Antioxidant, Hypolipidemic and Hypoglycaemic Compounds. Antioxidants.

[69] Biagi M., Noto D., Corsini M., Baini G., Cerretani D., Cappellucci G., Moretti E. (2019). Antioxidant Effect of the Castanea sativa Mill. Leaf Extract on Oxidative Stress Induced upon Human Spermatozoa. Oxidative Med. Cell. Longev..

[70] He S.-M., Chan E., Zhou S.-F. (2011). ADME Properties of Herbal Medicines in Humans: Evidence, Challenges and Strategies. Curr. Pharm. Des..

[71] Pradhan P.C., Saha S. (2015). Anthocyanin profiling of Berberis lycium Royle berry and its bioactivity evaluation for its nutraceutical potential. J. Food Sci. Technol..

[72] Hangun-Balkir Y., McKenney M.L. (2012). Determination of antioxidant activities of berries and resveratrol. Green Chem. Lett. Rev..

[73] Abdel-Tawab M. (2021). Considerations to Be Taken When Carrying Out Medicinal Plant Research—What We Learn from an Insight into the IC_50_ Values, Bioavailability and Clinical Efficacy of Exemplary Anti-Inflammatory Herbal Components. Pharmaceuticals.

[74] Cuomo J., Appendino G., Dern A.S., Schneider E., McKinnon T.P., Brown M.J., Togni S., Dixon B.M. (2011). Comparative Absorption of a Standardized Curcuminoid Mixture and Its Lecithin Formulation. J. Nat. Prod..

[75] Koeberle A., Werz O. (2014). Multi-target approach for natural products in inflammation. Drug Discov. Today.

[76] Dehghani F., Jandaghi S.H.S.S., Janani L., Sarebanhassanabadi M., Emamat H., Vafa M. (2021). Effects of quercetin supplementation on inflammatory factors and quality of life in post-myocardial infarction patients: A double blind, placebo-controlled, randomized clinical trial. Phytother. Res..

[77] Javadi F., Ahmadzadeh A., Eghtesadi S., Aryaeian N., Zabihiyeganeh M., Foroushani A.R., Jazayeri S. (2017). The Effect of Quercetin on Inflammatory Factors and Clinical Symptoms in Women with Rheumatoid Arthritis: A Double-Blind, Randomized Controlled Trial. J. Am. Coll. Nutr..

[78] Brüll V., Burak C., Stoffel-Wagner B., Wolffram S., Nickenig G., Müller C., Langguth P., Alteheld B., Fimmers R., Stehle P. (2017). No effects of quercetin from onion skin extract on serum leptin and adiponectin concentrations in over-weight-to-obese patients with (pre-) hypertension: A randomized double-blinded, placebo-controlled crossover trial. Eur. J. Nutr..

[79] Samsami-Kor M., Daryani N.E., Asl P.R., Hekmatdoost A. (2015). Anti-Inflammatory Effects of Resveratrol in Patients with Ulcerative Colitis: A Randomized, Double-Blind, Placebo-controlled Pilot Study. Arch. Med. Res..

[80] Brenjian S., Moini A., Yamini N., Kashani L., Faridmojtahedi M., Bahramrezaie M., Khodarahmian M., Amidi F. (2020). Resveratrol treatment in patients with polycystic ovary syndrome decreased pro-inflammatory and endoplasmic reticulum stress markers. Am. J. Reprod. Immunol..

[81] Khojah H.M., Ahmed S., Abdel-Rahman M.S., Elhakeim E.H. (2018). Resveratrol as an effective adjuvant therapy in the management of rheumatoid arthritis: A clinical study. Clin. Rheumatol..

[82] Bertelli A., Mannari C., Santi S., Filippi C., Migliori M., Giovannini L. (2008). Immunomodulatory activity of shikimic acid and quercitin in comparison with oseltamivir (Tamiflu) in an “in vitro” model. J. Med. Virol..

[83] Goc A., Sumera W., Rath M., Niedzwiecki A. (2021). Phenolic compounds disrupt spike-mediated receptor-binding and entry of SARS-CoV-2 pseudo-virions. PLoS ONE.

[84] Liu X., Raghuvanshi R., Ceylan F.D., Bolling B.W. (2020). Quercetin and Its Metabolites Inhibit Recombinant Human Angiotensin-Converting Enzyme 2 (ACE2) Activity. J. Agric. Food Chem..

[85] Ramdani L.H., Bachari K. (2020). Potential therapeutic effects of Resveratrol against SARS-CoV-2. Acta Virol..

[86] Kandeil A., Mostafa A., Kutkat O., Moatasim Y., Al-Karmalawy A.A., Rashad A.A., Kayed A.E., Kayed A.E., El-Shesheny R., Kayali G. (2021). Bioactive Polyphenolic Compounds Showing Strong Antiviral Activities against Severe Acute Respiratory Syndrome Coronavirus 2. Pathogens.

[87] Mehany T., Khalifa I., Barakat H., Althwab S.A., Alharbi Y.M., El-Sohaimy S. (2021). Polyphenols as promising biologically active substances for preventing SARS-CoV-2: A review with research evidence and underlying mechanisms. Food Biosci..

[88] Jang M., Park Y.-I., Cha Y.-E., Park R., Namkoong S., Lee J.I., Park J. (2020). Tea Polyphenols EGCG and Theaflavin Inhibit the Activity of SARS-CoV-2 3CL-Protease In Vitro. Evid.-Based Complement. Altern. Med..

[89] Yuan W., Wang J., An X., Dai M., Jiang Z., Zhang L., Yu S., Huang X. (2021). UPLC-MS/MS Method for the Determination of Hyperoside and Application to Pharmacokinetics Study in Rat After Different Administration Routes. Chromatographia.

[90] Steensma A., Faassen-Peters M.A.W., Noteborn H.P.J.M., Rietjens I. (2006). Bioavailability of Genistein and Its Glycoside Genistin As Measured in the Portal Vein of Freely Moving Unanesthetized Rats. J. Agric. Food Chem..

[91] Wu F., Zhou Y., Li L., Shen X., Chen G., Wang X., Liang X., Tan M., Huang Z. (2020). Computational Approaches in Preclinical Studies on Drug Discovery and Development. Front. Chem..

[92] Epstein J., Sanderson I.R., MacDonald T.T. (2010). Curcumin as a therapeutic agent: The evidence fromin vitro, animal and human studies. Br. J. Nutr..

[93] Meng T., Xiao D., Muhammed A., Deng J., Chen L., He J. (2021). Anti-Inflammatory Action and Mechanisms of Resveratrol. Molecules.

